# The Fever Hospitals of the Metropolitan Asylums Board.—II
*Part I. appeared in The Hospital of December 11, p. 279.


**Published:** 1912-01-06

**Authors:** R. A. Birdwood

**Affiliations:** Medical Superintendent of the Park Hospital.


### THE FEVER HOSPITALS OF THE METROPOLITAN ASYLUMS BOARD.—II.*

By R. A. BIRDWOOD, M.A., M.D., M.R.C.S., Medical Superintendent of the Park Hospital.

The isolation of the patients has in a modern hospital to be supplemented by some restraint on the part of the staff. Infection may be carried out by nurses, laundrymaids and others in two ways. They may have a mild unrecognised attack of the disease when visiting their friends. This undoubtedly does happen, and most generally soon after joining, but it may happen after years of service. The other way is by the wearing of infected clothes. Both these sources of infection can be controlled by administrative methods. The importance of these modes of spread is apparent, for homes must be built for all employed in the hospital if they happen to come in contact with the patients. These homes must be within the hospital enclosure.

### General v. Isolation Hospitals.

Not only nurses' homes are required, but the laundry, kitchen, and needle-room staff must live in, and so must all the men brought into contact with the patients or their clothing. In this respect there is a great difference between general and isolation hospitals, for with us all the women staff and almost all the men reside on the hospital site. Their laundry, the preparation of their food, and attendance on them is all part of the hospital duties. For the same reason it is undesirable to bring in stranger workmen for repairs or other work; accordingly, men to do these works are retained. So constituted we are an isolated village community, nearly self-contained.

The justification for this is the infrequence of mishap in the families or amongst the friends of the staff, attributable to communication with the hospital workers. The preventive work of the fever hospital would be incomplete unless some means were available for the conveyance of the infectious sick from their homes to the hospital. The ambulance service of the Metropolitan Asylums Board secures, on application, the patient's immediate removal in comfort and safety. On the receipt of a medical certificate that it is desirable to remove to hospital anyone suffering from one of the isolable diseases a horsed or motor conveyance, with stretcher, clothes, and refreshment for the journey leaves the ambulance station. A nurse is always taken, and a man attendant if the patient is over 12 years of age. So that there is no need for anyone other than the hospital staff to come in contact with the patient when being taken from the bedroom to the coach. In case of any emergency on the way the nurse is instructed and authorised how to act. Most Londoners have some knowledge of the ambulance work of the Board; those who have had personal experience are appreciative, as its excellence is indisputable.

We have then in early notification, in rapid and safe removal from populous districts, and in voluntary segregation and free treatment for everyone who is willing to accept it, the modern weapons for the control of epidemics.

Most of the patients admitted are children, except in the case of admissions for enteric fever. The stay in hospital after recovery and before fitness for discharge from hospital is irksome to adults. It is difficult to provide congenial occupations for grown-up fever convalescents. But these illnesses do impair the patients' strength, and their loss of vigour is much greater than they at first realise. In such circumstances enforced idleness is in many instances an unmitigated boon and the best treatment for enabling the man or woman to recuperate and fitting them for their ordinary occupations. An earlier return to work would sometimes result in a physical breakdown.

With convalescent children it is otherwise. It is easy enough to keep them occupied and entertained where only violent exercise is forbidden. In innumerable instances the happiest times of their lives and their pleasantest memories are associated with their stay in one of the Board's hospitals. One can hardly doubt that amongst the influences tending to the amelioration of our social conditions the experience of these little people in cleanliness, order, and discipline must have some lasting effect.

Probably the tendency in recent times has been to prolong the patient's average stay in hospital. Last year it averaged for recovered scarlet-fever patients from the London hospitals of the Board 63 days, and for those from the country hospitals ten days longer. For diphtheria the stay was 57 and 77 days for town and country discharges respectively. From these figures it may be gathered that the provision of suitable occupation during convalescence forms no small part of an isolation hospital's work. It may be said that during the whole of the time the patient's liability to the occurrence of the complications and sequelæ incidental to these diseases makes it desirable that these little patients should be kept under constant professional and nursing supervision.

Wherever the environment outside the hospital is unfavourable to good recovery after illness the full advantage of hospital treatment during that illness cannot be obtained unless the child is detained till restored to health: indeed we might consider the effort to save as being almost wasted if the enfeebled sufferer is sent away too soon. In the future possibly the average stay in hospital will be still further prolonged in order that the maximal value may be obtained for those most in need of it. It is to be hoped that at some not far distant date the Board will decide to send their fever convalescent children to homes at the seaside. If proof is required that our ancestors were fisherfolk, surely the well-known rapid recovery of such children on the seashore might be regarded as conclusive demonstration of our seafaring origin.

* Part I. appeared in The Hospital of December 11, p. 279.

A Grave Misconception.

It is often suggested that work in a fever hospital is monotonous and uninteresting for the medical officers and nurses; that the inherent risks are not compensated by knowledge and skill acquired by intimate acquaintance of the diseases and their healing. There can be no graver misconception. No general practitioner should consider himself fully equipped to advise and treat unless he is fairly familiar with the ordinary manifestations of these infectious diseases and the administrative control of epidemics. Nor should any trained nurse undertake the care of a seriously ill person suffering from a severe attack unless she has had experience in our work.

The Interest of Fever Work.

It would be of the greatest assistance to both to spend six months or a year with us. Not only would they add to their stock of knowledge, but they would be fascinated with their opportunity of doing good. In the healing art the interest comes when we know that our efforts are rewarded by lives saved or prolonged or made more endurable. Perhaps of no other forms of sickness can it be said that so much depends on the nurse and the doctor in determining the ultimate recovery not only with life but with health, sight, hearing, or without disfigurement and the loss of all that we value in physical fitness. At any rate, workers in fever hospitals do find the work not only interesting but worth doing well, especially after they have recovered from the initial repulsion quite natural to most of us.

As there is much to be learnt and more to be done with comparatively few to do it, may I be permitted to advise all those at the threshold of their professional careers, whether it be nursing or medicine, to devote at least a year, if they can, by holding an appointment in a fever hospital. Great as are the advantages of isolation, it must be acknowledged that there are drawbacks. Designed to prevent the spread of disease, fever hospitals are the means of spread in three ways. These are spoken of as "errors of diagnosis," "return cases," and "secondary infections." There are many conditions bearing a close resemblance to the manifestations of the exanthemata, making it difficult to diagnose between infectious and non-infectious diseases. The mistake is found out when after admission the patient develops the other disease.

Observation Wards.

To meet this difficulty large numbers of observation wards of one or two beds in each are provided in the more recently built hospitals for those patients presenting uncertainty of diagnostic symptoms on admission or later. Some think (there is much to be advanced in support) that many of these mishaps would be prevented if the services of the experienced medical staff could be secured by the general practitioner in consultation when he was in doubt about the diagnosis and how to act about removal from home. Possibly this may come about in time; one wonders why it has not already. Sometimes the exanthemata resemble each other, and the wrong disease may be admitted into a ward, to be followed by an outbreak. Or a patient with one infectious disease may be incubating another at the time of admission. Beds must be set apart to deal effectively with such outbreaks, and the vigilance of the staff in detecting the earliest manifestations of the invader may prevent an extensive outbreak. The third trouble is known as "return cases," the greatest anxiety of all. The term "return case" is applied to any member of the patient's household or other person coming into contact with the recovered patient, soon after discharge from hospital, if such relative or friend is infected in consequence of the contact—in short, that the patient was discharged in an infectious condition. The solution of this problem is not yet in sight. We do know that these cases are not the result of premature discharge; we do not know why it occurs with some and not with others. No effort has been spared by the managers to try and find out by the methods of investigation how this can be prevented.

Risk of Moral Contamination.

When that has been discovered one great step forward will have been taken. These are three physical disadvantages, but there is one that outweighs them all with many parents. It is said that there is some contamination of innocent children as a result of association with the vicious—that they have learnt bad language or acquired rude manners by their stay in hospital. Let it be admitted that it does occur. I am sure it does not do so to any great extent. The evil is present. That it has never taken strong root is due to the vigilance and influence of the many good women who are constantly present as nurses in our wards. There is much less trouble about this now than there used to be years ago. Soon it will be less or absent altogether. After all, if the bad do the good some harm, we have the reciprocal action of the good doing the bad some good. It only means that there must be no mistake about putting the right sort of woman to preside over each hospital unit, the ward.

The Need for Research.

Another charge, most likely a just charge, brought against our work is that whilst we alone have the clinical opportunity of study from the collection of such large numbers of patients, we have made no great advance in the knowledge of our diseases; that although we are teaching institutions professing to give instruction, we do little or nothing by research methods to find out either causation, prevention, or cure. We have not done systematic research work, and the Asylums Board ought to do it. The sooner it is begun the better. If by spending £10,000 a year on research (by that is meant the employment of men capable of doing it) an expenditure of ten times that amount on the maintenance of patients could be prevented, then the whole gain would be the community's. A pathological department of the M.A.B. worthy of the metropolis is the immediate need and its absence the hiatus of isolation administraton.

